# EGFR-mutated lung adenocarcinoma presenting as miliary intrapulmonary carcinomatosis: A diagnostic mimic of tuberculosis

**DOI:** 10.1016/j.rmcr.2026.102502

**Published:** 2026-10-02

**Authors:** Gianpiero Paletta, Srujan Kancharla, Alexis Magre, Naveen Tyagi

**Affiliations:** a Texas A&M University Naresh K. Vashisht College of Medicine, Round Rock, TX, USA; b Baylor Scott & White Medical Center, Round Rock, Round Rock, TX, USA

## Abstract

Miliary intrapulmonary carcinomatosis (MIPC) is a rare metastatic pattern of lung cancer that closely mimics disseminated tuberculosis (TB), posing a significant diagnostic challenge. We present a 53-year-old Filipino woman, a lifelong nonsmoker with prior latent TB, who presented with progressive cough and a diffuse bilateral miliary nodular pattern on chest imaging. An extensive infectious workup was unrevealing, and bronchoscopic biopsy revealed poorly differentiated adenocarcinoma (TTF-1+) harboring an EGFR exon 21 L^85^8R mutation. Staging demonstrated brain, osseous, and pleural metastases (stage IVB NSCLC). The patient was initiated on osimertinib 80 mg daily with palliative radiation and zoledronic acid, achieving no evidence of active disease at 2.5 years on serial PET-CT and brain MRI. This case underscores the importance of tissue diagnosis in unresolved miliary patterns, early molecular profiling in lung adenocarcinoma, and the potential for durable exceptional responses with osimertinib in EGFR-mutated NSCLC.

## 1 Introduction

Miliary intrapulmonary carcinomatosis (MIPC) is a rare manifestation of metastatic lung cancer characterized by diffuse pulmonary micronodules. This is reported in approximately 2.4% of lung cancer cases and is most frequently associated with EGFR-mutated tumors [1,2]. A miliary pattern on imaging closely resembles disseminated tuberculosis (TB), and patients frequently undergo extensive infectious evaluation prior to diagnosis; however, this imaging pattern carries a broad differential diagnosis, including disseminated infections, fungal disease, sarcoidosis, hypersensitivity pneumonitis, and silicosis, and often presents a significant diagnostic challenge [1]. Approximately two-thirds of non-small cell lung cancer (NSCLC) patients present with advanced disease at initial diagnosis, with a median survival time of four to five months and a one-year survival rate of 10% when managed with best supportive care alone [1]. A case-control study from China noted that some cohort studies have reported the incidence of cancer in tuberculosis (TB) patients to range from 7.2 to 11.6% [3].

NSCLC harboring epidermal growth factor receptor (EGFR) mutations represents a distinct molecular subset, more commonly observed in individuals of Asian ancestry and in never-smokers [2]. Historically, metastatic NSCLC presenting with diffuse pulmonary disease carried a poor prognosis. However, the identification of EGFR mutations has transformed treatment, with EGFR tyrosine kinase inhibitors such as osimertinib significantly improving survival and demonstrating central nervous system activity, as seen in the FLAURA phase III trial [4]. We report a case of EGFR exon 21–mutated lung adenocarcinoma presenting as miliary intrapulmonary carcinomatosis in a patient with previously treated latent tuberculosis, who had an excellent response to tyrosine kinase inhibitor therapy. Beyond reinforcing the recognized association between EGFR mutations and a miliary imaging pattern, this case highlights an important diagnostic mimic of disseminated tuberculosis and illustrates an exceptionally durable complete response to first-line osimertinib monotherapy in high-burden L858R disease.

## 2 Case presentation

A 53-year-old woman with a past medical history of latent tuberculosis treated with six months of isoniazid presented to her primary care physician with a chronic nonproductive cough. Four months prior, she reported a COVID-19 infection associated with cough that subsequently resolved. The cough gradually returned and was accompanied by night sweats and unintentional weight loss, which she attributed to perimenopause. She denied fever or chills and was a lifelong nonsmoker. She had immigrated from the Philippines more than 30 years earlier.

A chest radiograph (Fig. 1a) obtained in the primary care setting demonstrated diffuse pulmonary micronodules concerning for miliary tuberculosis, prompting referral to the emergency department. On admission, laboratory evaluation was notable for an elevated alkaline phosphatase level (509 IU/L), consistent with her subsequently identified osseous metastatic burden, with otherwise unremarkable results. Serum tumor markers, including carcinoembryonic antigen (CEA), were not obtained during this admission. Computed tomography (CT) of the chest revealed a dominant left upper lobe nodule with innumerable miliary nodules, a small left pleural effusion with nodular pleural thickening, and multiple sclerotic osseous lesions (Fig. 1b).

**Fig. 1:**
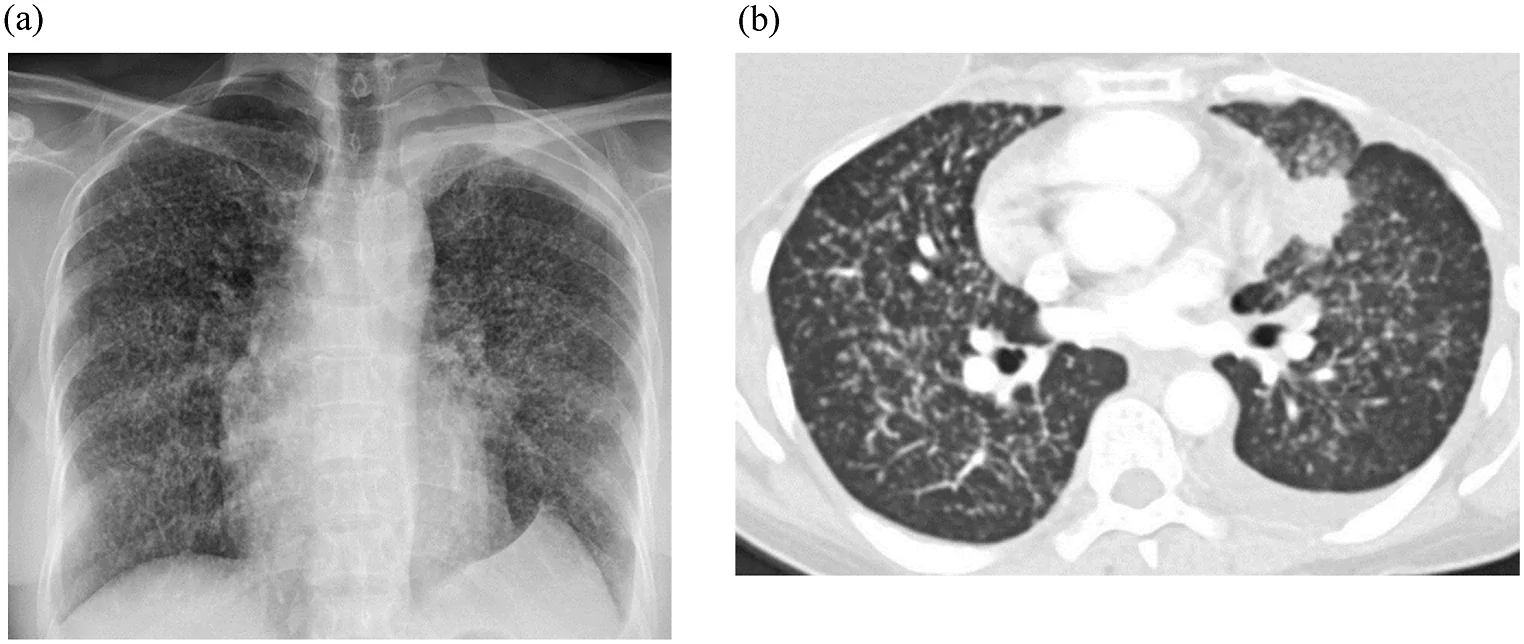
(a) Radiograph of the chest, AP view, showing diffuse pulmonary micronodules. (b) CT of the chest, axial view, showing diffuse pulmonary micronodules and a dominant left upper lobe nodule.

An extensive infectious evaluation, including three sputum acid-fast bacilli smears, blood cultures, tuberculosis polymerase chain reaction, and QuantiFERON-TB Gold testing, was negative. Given persistent concern for malignancy, the patient underwent bronchoscopy with visual airway inspection of the tracheobronchial tree including the right upper lobe, right middle lobe, right lower lobe, left upper lobe, lingula, and left lower lobe. An endobronchial mass like lesion was noted at the lingula entry/takeoff, with forceps biopsy being performed. Bronchoalveolar lavage of the RUL and RLL was then obtained. Transbronchial biopsies was then performed at RLL superior segment. The dominant left upper lobe mass was not directly sampled because of malignant appearance of the endobronchial lesion mentioned above. Pathologic analysis of the RLL/transbronchial biopsy demonstrated bronchial mucosa and alveolated lung parenchyma with no significant histopathology. The lingula/endobronchial lesion demonstrated poorly differentiated adenocarcinoma. Immunohistochemistry was positive for TTF-1, and molecular testing identified an EGFR exon 21 L^85^8R mutation, with no BRAF or KRAS mutations identified. The patient was discharged with plans for outpatient oncology follow-up.

One week after discharge, positron emission tomography–computed tomography (PET-CT) demonstrated numerous hypermetabolic osseous metastatic lesions, a left pleural effusion with hypermetabolic pleural thickening, and hypermetabolic left hilar and mediastinal lymphadenopathy (see Fig. 2). Magnetic resonance imaging (MRI) of the brain revealed at least five subcentimeter enhancing metastatic foci throughout the supratentorial and infratentorial regions, and she remained asymptomatic despite these brain metastases. She subsequently underwent palliative radiation therapy (30 Gy in 10 fractions) to pelvic and sacral metastatic lesions and achieved excellent pain control. She was also started on zoledronic acid for the osseous metastases. Concurrently, she was initiated on targeted therapy with osimertinib 80 mg orally once daily. She showed a good response one month after starting osimertinib, as seen on repeat brain MRI. After six months of therapy, with regular PET/CT and brain MRI surveillance, she showed no hypermetabolic activity on PET/CT and no active disease on brain MRI. Her pain medication requirements declined during this period.

**Fig. 2:**
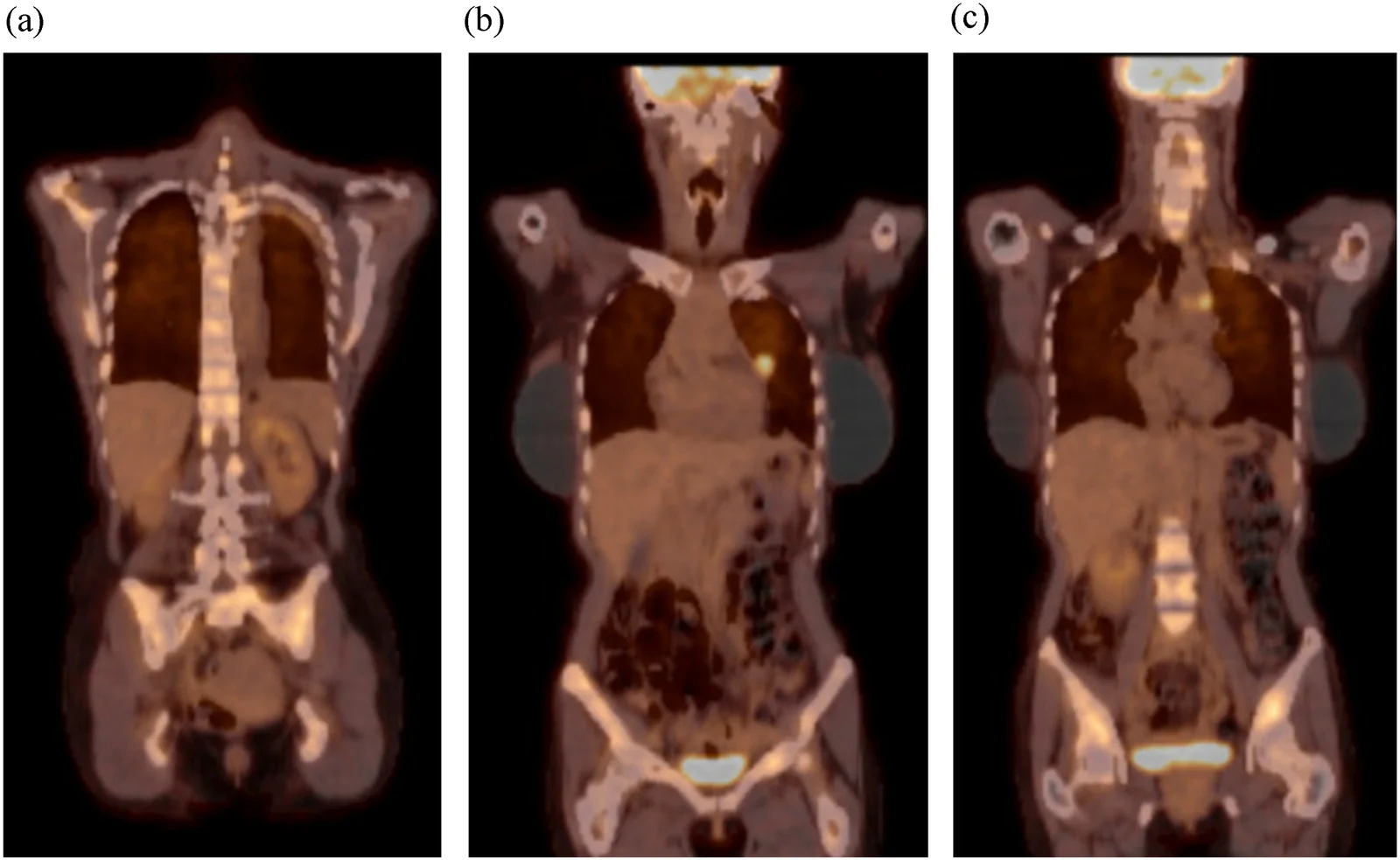
(a) PET/CT showing multiple lesions within the osseous pelvis and numerous lesions throughout the spine. (b) A hypermetabolic mass measuring 2.3 × 2.1 cm in the left upper lobe abutting the pericardium. (c) Hypermetabolic left hilar lymph nodes.

At two and a half years, serial imaging demonstrated no evidence of active metastatic disease. The patient remains functionally active, continues to take osimertinib, recently completed a 5-km walk with her children, and has not required pain medications for several months.

## 3 Discussion

This patient had an unusual presentation of metastatic NSCLC, with a prior history of treated latent TB. Because the reported incidence of cancer in tuberculosis (TB) patients ranges from 7.2 to 11.6%, malignancy should remain on the differential in patients with TB. This patient underwent an extensive workup for TB during admission; however, three sputum acid-fast bacilli smears, blood cultures, tuberculosis polymerase chain reaction, and QuantiFERON-TB Gold testing were negative. She subsequently underwent bronchoscopy with endobronchial and transbronchial biopsies, which were diagnostic. Miliary nodules on imaging commonly lead to an extensive workup for TB, but awareness of malignant etiologies is critical when infectious studies remain negative. This pattern has a broad differential, including bacterial and fungal infections, immune-mediated processes such as hypersensitivity pneumonitis and sarcoidosis, and malignant processes, as in this case of EGFR-positive NSCLC [2,5]. The mechanism underlying miliary spread in EGFR-mutated lung adenocarcinoma is not fully understood but is thought to reflect hematogenous dissemination of tumor emboli through the pulmonary microvasculature, producing diffuse micronodular metastases throughout the lungs [6]. EGFR-mutant adenocarcinomas are disproportionately represented among NSCLC presenting in a miliary distribution, and this enrichment suggests that the pattern is biologically linked to EGFR-driven disease; accordingly, a miliary presentation in a never-smoker should itself prompt molecular testing for EGFR [1,2]. In addition, she had TTF-1 positivity, which supports a pulmonary origin for the adenocarcinoma, as this immunohistochemical marker is commonly expressed in lung adenocarcinomas and is widely used to distinguish primary lung tumors from metastatic adenocarcinomas [7].

Distinguishing MIPC from miliary tuberculosis on imaging is challenging, as both produce randomly distributed bilateral micronodules and no single radiographic feature is diagnostic [8]. Certain features may favor one diagnosis over the other. Miliary tuberculosis classically produces uniform 1–3 mm nodules of relatively even size and may be accompanied by tree-in-bud opacities, cavitation, calcified granulomas, or low-attenuation necrotic mediastinal lymphadenopathy. Metastatic micronodules, including MIPC, tend to be larger and more variable in size and are more frequently associated with interlobular septal and peribronchovascular thickening and with non-necrotic lymph node enlargement [6]. In our patient, the combination of a dominant left upper lobe mass, variably sized nodules, nodular pleural thickening with effusion, and hypermetabolic hilar and mediastinal lymphadenopathy favored a malignant rather than an infectious process. Furthermore, the relatively indolent clinical picture—chronic cough without fever or chills and asymptomatic brain metastases—was atypical for disseminated infection and further raised suspicion for malignancy. Because imaging overlap is substantial, tissue confirmation remains essential when infectious studies are unrevealing.

Lung adenocarcinoma is the most common subtype globally and accounts for 60–80% of cases in never-smoker individuals [5]. Prior tuberculosis is a lesser-recognized, smoking-independent risk factor for lung cancer; a recent systematic review and meta-analysis identified an association between prior lung diseases, including tuberculosis, and lung cancer specifically among individuals with no smoking history, and a large population-based cohort of tuberculosis survivors found previous tuberculosis to be an independent risk factor for lung cancer regardless of smoking status [9,10]. The proposed mechanism involves chronic inflammation, fibrosis, and parenchymal scarring that promote local carcinogenesis. This evidence derives predominantly from cohorts with prior active (pulmonary) tuberculosis rather than latent tuberculosis infection; our patient had latent tuberculosis treated with isoniazid, without documented prior active pulmonary disease, so this association is best regarded as a possible rather than a definitive contributor in her case. Obtaining an in-depth social and infectious history is important for risk stratification. Although TB remains relatively uncommon in the United States, recent surveillance data show rising case counts, with the majority of cases occurring among individuals born outside the United States or those with exposure to high-incidence regions through travel or migration [11,12].

Only 2.4% of NSCLC patients present with MIPC at the time of diagnosis, but among these patients, 70% harbor an EGFR mutation [1,2]. EGFR is the second most mutated oncogene in NSCLC and the most common in never-smoker and Asian populations [5,13]. The identification of EGFR driver mutations has reshaped therapeutic strategies in NSCLC, with third-generation EGFR tyrosine kinase inhibitors, namely osimertinib, demonstrating superior survival outcomes and enhanced central nervous system activity in the FLAURA phase III trial [4].

This was particularly evident in our patient, who had multiple metastatic lesions throughout the supratentorial and infratentorial brain (Fig. 3a and b). With prompt administration of osimertinib, she had no active disease on brain MRI and no hypermetabolic activity on PET/CT six months after starting treatment. Understanding the association between a MIPC presentation in NSCLC and EGFR mutation is important for choosing the correct targeted therapy. EGFR tyrosine kinase inhibitors (TKIs) have become the standard of care for EGFR-positive NSCLC, particularly the third-generation agent osimertinib, which has superior CNS activity and improves overall survival [4,14].

**Fig. 3:**
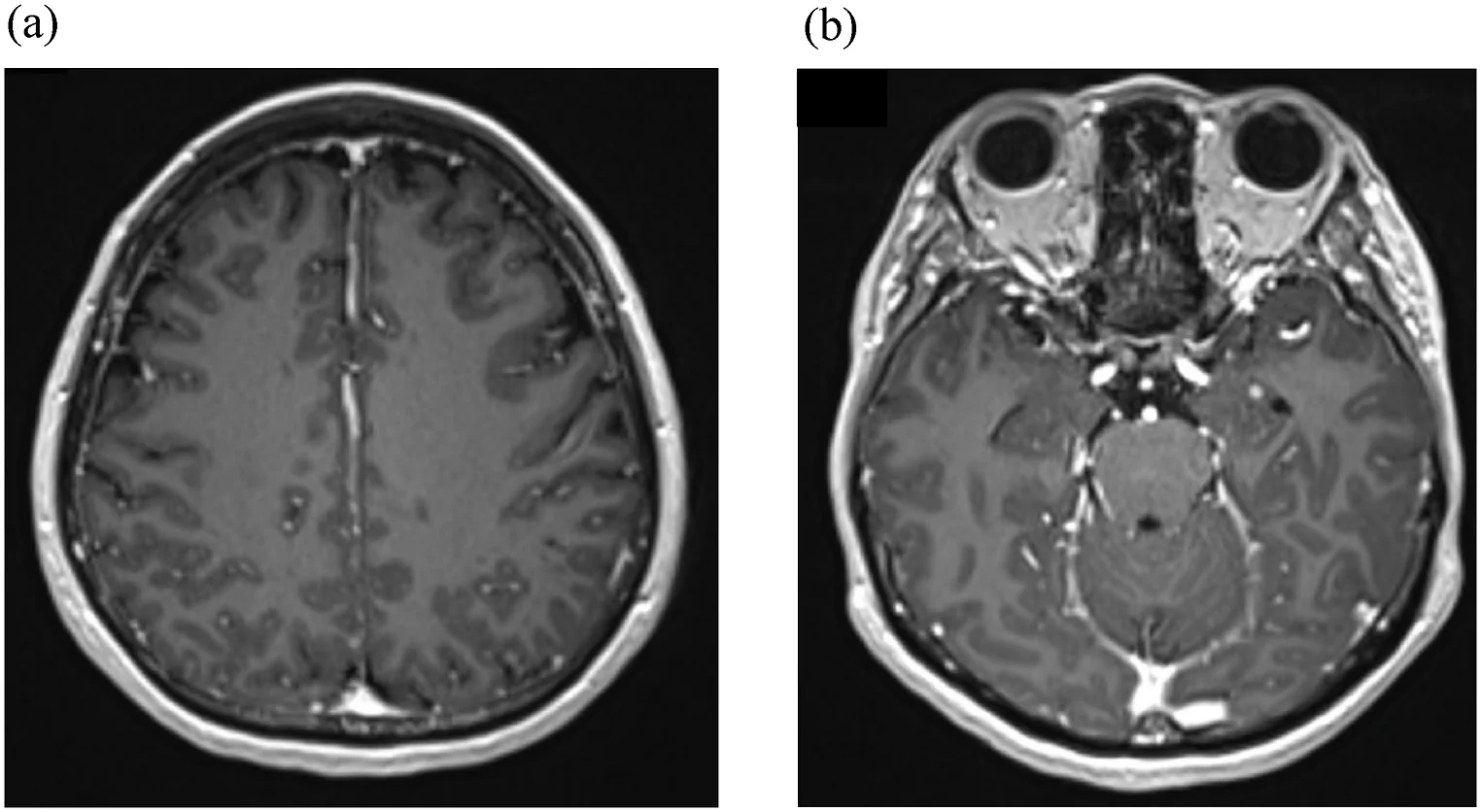
(a, b) Initial brain MRI showing at least five subcentimeter enhancing metastatic foci throughout the supratentorial and infratentorial brain.

Our findings are consistent with prior reports linking a miliary presentation to EGFR-mutant disease. Wu et al. found a high frequency of EGFR mutations among patients with NSCLC presenting as miliary intrapulmonary carcinomatosis [2], and Kim et al. similarly reported that lung adenocarcinomas with miliary disseminated carcinomatosis were enriched for EGFR mutations and frequently accompanied by brain metastases [1]. Like these cohorts, our patient was an Asian never-smoker with EGFR-mutant adenocarcinoma, brain metastases, and a diffuse miliary pattern. She is distinguished, however, by the depth and durability of her response—no evidence of active disease at 2.5 years on osimertinib—which exceeds the historically guarded outcomes associated with miliary EGFR-mutant disease.

First-line systemic therapy for EGFR-mutated NSCLC continues to evolve. At the time of our patient's diagnosis, osimertinib monotherapy was the established first-line standard based on the FLAURA trial [4]. More recently, the FLAURA2 trial demonstrated that adding platinum–pemetrexed chemotherapy to osimertinib significantly prolongs overall survival compared with osimertinib alone (median 47.5 vs 37.6 months), with benefit retained in patients with L858R mutations and in those with baseline central nervous system metastases—both features present in our patient [15]. Anti-angiogenic and other combination strategies are likewise increasingly considered for patients with high disease burden, and the L858R mutation has generally been associated with less favorable tyrosine kinase inhibitor outcomes than exon 19 deletions. Against this backdrop, our patient's sustained complete response to single-agent osimertinib, despite extensive osseous, pleural, and intracranial disease, represents an exceptional and durable outcome rather than the expected course.

This report is limited by its single-patient design and by the absence of serum tumor marker data, including CEA, which were not obtained during the patient's evaluation and might otherwise have aided diagnosis and monitoring.

### 3.1 Learning points

1. A miliary pattern on chest imaging has a broad differential diagnosis, including infectious, inflammatory, and malignant etiologies, which may present with overlapping clinical features
2. Prior tuberculosis, particularly prior active pulmonary tuberculosis, is a lesser-recognized risk factor for lung cancer independent of smoking history; the contribution of latent tuberculosis infection alone is less well established.
3. A miliary pattern of lung cancer is concerning for EGFR-positive adenocarcinoma, and early molecular testing is essential because targeted therapy can significantly improve outcomes

## 4 Conclusion

This case underscores the importance of maintaining a broad differential diagnosis when evaluating chronic cough with a miliary radiographic pattern. After exclusion of infectious and inflammatory causes, tissue diagnosis is essential to confirm malignancy. Recognition of tuberculosis as an independent risk factor for lung cancer, together with the association between a miliary pattern and EGFR-mutated NSCLC, allows timely initiation of targeted therapy, which may significantly improve systemic and intracranial disease control.

Although disseminated tuberculosis is often the leading consideration with a miliary pattern on imaging, particularly in patients from endemic regions or with prior TB exposure, malignant etiologies such as metastatic lung adenocarcinoma must also be considered. Failure to recognize this possibility may lead to delays in diagnosis and appropriate oncologic therapy. Early consideration of malignancy and timely tissue diagnosis are essential, as identification of actionable mutations such as EGFR can dramatically alter prognosis through targeted therapies.

## CRediT authorship contribution statement

**Gianpiero Paletta:** Writing – review & editing, Writing – original draft. **Srujan Kancharla:** Writing – review & editing. **Alexis Magre:** Writing – review & editing. **Naveen Tyagi:** Supervision.

## Declaration of competing interest

The authors declare that they have no known competing financial interests or personal relationships that could have appeared to influence the work reported in this paper.
